# A nurse-led rheumatology clinic versus rheumatologist-led clinic in monitoring of patients with chronic inflammatory arthritis undergoing biological therapy: a cost comparison study in a randomised controlled trial

**DOI:** 10.1186/s12891-015-0817-6

**Published:** 2015-11-16

**Authors:** Ingrid Larsson, Bengt Fridlund, Barbro Arvidsson, Annika Teleman, Petra Svedberg, Stefan Bergman

**Affiliations:** School of Health and Welfare, Halmstad University, Box 823, S-30118 Halmstad, Sweden; Spenshult Research and Development Centre, Halmstad, Sweden; School of Health and Welfare, Jönköping University, Jönköping, Sweden; Capio Movement Hospital, Halmstad, Sweden; Primary Health Care Unit, Department of Public Health and Community Medicine, Institute of Medicine, University of Gothenburg, Gothenburg, Sweden

**Keywords:** Biological therapy, Chronic inflammatory arthritis, Cost comparison, Person-centred care, Nurse-led rheumatology clinic, Randomised controlled trial

## Abstract

**Background:**

Recommendations for rheumatology nursing management of chronic inflammatory arthritis (CIA) from European League Against Rheumatism (EULAR) states that nurses should take part in the monitoring patients’ disease and therapy in order to achieve cost savings. The aim of the study was to compare the costs of rheumatology care between a nurse-led rheumatology clinic (NLC), based on person-centred care (PCC), versus a rheumatologist-led clinic (RLC), in monitoring of patients with CIA undergoing biological therapy.

**Methods:**

Patients with CIA undergoing biological therapy (*n* = 107) and a Disease Activity Score of 28 ≤ 3.2 were randomised to follow-up by either NLC or RLC. All patients met the rheumatologist at inclusion and after 12 months. In the intervention one of two annual monitoring visits in an RLC was replaced by a visit to an NLC. The primary outcome was total annual cost of rheumatology care.

**Results:**

A total of 97 patients completed the RCT at the 12 month follow-up. Replacing one of the two annual rheumatologist monitoring visits by a nurse-led monitoring visit, resulted in no additional contacts to the rheumatology clinic, but rather a decrease in the use of resources and a reduction of costs. The total annual rheumatology care costs including fixed monitoring, variable monitoring, rehabilitation, specialist consultations, radiography, and pharmacological therapy, generated €14107.7 per patient in the NLC compared with €16274.9 in the RCL (*p* = 0.004), giving a €2167.2 (13 %) lower annual cost for the NLC.

**Conclusions:**

Patients with CIA and low disease activity or in remission undergoing biological therapy can be monitored with a reduced resource use and at a lower annual cost by an NLC, based on PCC with no difference in clinical outcomes. This could free resources for more intensive monitoring of patients early in the disease or patients with high disease activity.

**Trial registration:**

The trial is registered as a clinical trial at the ClinicalTrials.gov (NCT01071447). Registration date: October 8, 2009.

## Background

Chronic inflammatory arthritis (CIA) mainly refers to rheumatoid arthritis (RA) and the group of spondyloarthritis (SpA), including ankylosing spondylitis and psoriatic arthritis [1]. The primary goal of CIA treatment is to suppress disease activity by control of the inflammation in order to achieve remission or low disease activity as well as to prevent joint damage and early death [2, 3]. Disease activity and inflammation in patients with CIA have declined over the past decade since the introduction of biological therapy [4]. Previous research has demonstrated that biological therapies lead to a reduction in disease activity and radiological progression [5], better health status and higher level of quality of life [6]. The biological therapies have a high impact on the immune system and require regular monitoring every 6–12 months even when patients have achieved low disease activity or remission [3].

Living with CIA affects patients’ physical functioning but also emotional, psychological and social aspects that in turn have a global impact on the whole life situation [7]. The key element of advanced nurse-led clinics (NLC) is a holistic approach including person-centred care (PCC). PCC involves patients as partners in care, and integrates teamwork [8]. The PCC is advocated by the WHO as a key component of quality healthcare [9]. Previous research has demonstrated that PCC is a way for increasing satisfaction with care both for patients [10] and nurses [11]. PCC also leads to improved health outcomes and reduces the length of a hospital stay with no negative impact on health-related quality of life [12, 13]. A systematic review reported good effectiveness of tight control at an NLC in patients undergoing conventional Disease-Modifying Anti-Rheumatic Drug (DMARD) therapy [14]. Recent research showed similar results, with increased patient satisfaction [15–17] and lower consultation costs at an NLC than at a rheumatologist-led clinic (RLC) [17, 18]. Two decades after introduction of biological therapy the consumption of inpatient and outpatient care has decreased but the total direct costs have increased due to the cost of biological therapy [19].

The recommendations of the European League Against Rheumatism (EULAR) about the role of the nurse in the management of CIA, emphasize that nurses can contribute to cost savings in rheumatology care through interventions and monitoring as part of a comprehensive disease management [1]. An NLC based on PCC in monitoring biological therapy in patients with stable CIA is a way of meeting the EULAR recommendations and an opportunity for achieving cost savings.

In order to fill a knowledge gap regarding NLC in monitoring biological therapy we conducted a randomised controlled trial (RCT) with a 12 month follow-up [20]. The hypothesis was that treatment outcome as measured by the Disease Activity Score 28 (DAS28) in patients with low disease activity or in remission, whose biological therapy was monitored at the NLC, based on PCC, would not be inferior to that obtained at a rheumatologist-led clinic (RLC). There were no differences in the changes in the DAS28 (*p* = 0.66) or Health Assessment Questionnaire (HAQ) (*p* = 0.79) between an NLC or an RLC [20]. A complementary qualitative approach showed that the NLC provided added value to the patients by providing a sense of security, familiarity and participation [21]. It is of interest to evaluate differences in resources and costs when replacing RLC by NLC in the monitoring of biological therapy.

Based on the previous RCT, the aim of this study was thus to compare the use of resources and costs of rheumatology care between an NLC, based on PCC, versus an RLC, in monitoring of patients with CIA undergoing biological therapy.

## Methods

### Study design and setting

This is a cost comparison study, based on an RCT, where the monitoring of biological therapy by a rheumatology nurse is compared with that of a rheumatologist. The RCT took place at rheumatology clinic in southern Sweden with 5500 outpatient visits annually by 3500 patients, of whom 600 received biological therapy. The clinical outcomes of the study has previously been published [20, 21]. Use of resources and costs, including the patients’ all contacts at the rheumatology clinic, were recorded prospectively. A review of the patients’ medical records was performed to validate the data recorded during the study.

### Participants

Patients over 18 years with CIA with an ongoing biological therapy and a DAS28 ≤ 3.2 were eligible for the RCT. Patients with RA, undifferentiated arthritis (UA), undifferentiated spondyloarthritis (USpA) and psoriatic arthritis (PsA) were included in the trial if they had a history of peripheral arthritis. Exclusion criteria were patients with recurrent infections or adverse events due to the biological therapy or unwillingness to be monitored at the NLC. Between October 2009 and August 2010 patients were assessed by a rheumatologist at their usual monitoring visit to establish if they were eligible to participate in a well-powered RCT [20]. All patients met a rheumatologist at inclusion and after 12 months and were, in the intervention group, monitored by a rheumatology nurse after 6 months. If necessary, the nurses could contact the rheumatologist for advice or to obtain a prescription. The patients in the RCL (the control group) were monitored by their rheumatologist every 6 months as usual.

### Intervention

An NLC, based on PCC was designed to monitor patients’ biological therapy. After randomisation to the NLC, patients received information about contact details to their rheumatology nurse, and that they when needed could contact their nurse during the 12 months study period. In the intervention one of two annual monitoring visits at the RCL was replaced by a visit to the NLC. The nurses assessed the patients’ disease activity by examining tender and swollen joints based on the 28-joint count in addition to evaluating the results of laboratory test. Drug treatment was discussed in terms of administration, adherence, side effects and blood samples as well as patients’ global health. Data were stored in the Swedish Rheumatology Quality Register (SRQ) [22]. In addition to assessing patients’ disease activity the visit at the NLC focused on the patient’s needs at that specific moment. PCC focuses on patients’ resources and abilities to manage their lives and patients are seen as experts in their illness and life situation [23]. The nurses listened sensitively to the patient’s illness narrative which, together with the symptoms of the disease, provided the nurse with a good foundation for discussing and planning care and treatment together with the patient. Five registered nurses with 9–20 years’ experience of managing rheumatic diseases had undergone special training from a rheumatologist and RA instructors to assess swollen and tender joints based on the 28-joint count. The latter were specially trained patients who instruct healthcare staff how to examine the joints of the hands, wrists, feet and ankles as well as providing information about living with the disease. The monitoring visits at both the NLC and RLC lasted for 30 min including time for administration and documentation as usual at the rheumatology outpatient clinic.

### Primary outcome measure

The primary outcome measure was the total annual use of resources and direct costs of rheumatology care in monitoring biological therapy during the 12 month period. This included costs for fixed monitoring, variable monitoring, rehabilitation, specialist consultations, radiography, and pharmacological therapy. The costs for monitoring resources varied over the study period 2009 to 2011 due to changes in the established tariffs for rheumatology care in the region of southern Sweden where the study was conducted. All costs were calculated in the actual prices when the resources were used in the study. The costs were converted from Swedish kronor (SEK) to euros at the rate of 1 SEK = €0.11 which was the exchange rate of euro vs. SEK when the study was completed in August 2011.

### Secondary outcome measures

Secondary outcome measures were the annual use of resources and direct costs for fixed monitoring, variable monitoring, rehabilitation, specialist consultations, radiography, and pharmacological therapy, representing the different parts of the primary outcome. Fixed monitoring resources and costs included: a monitoring visit at 6 months to a rheumatology nurse (€ 115.0 to 117.4) or to a rheumatologist (€ 232.1 to 237.0), for both groups a monitoring visit at 12 months to a rheumatologist (€ 232.1 to 237.0), and monitoring blood tests (€ 15.4 to 15.5). Variable monitoring resources and costs included: additional telephone calls to a rheumatology nurse (€6.2 to 6.4), additional telephone calls to a rheumatologist (€16.9 to 17.2), additional rheumatologist visits (€ 232.1 to 237.0), cortisone injections in addition to regular rheumatologist monitoring visits (€174.1 to 177.7), and additional blood tests (€1.2 to 30.7). Rehabilitation resources and costs included: team rehabilitation days of care in inpatient (€406.2 to 414.8) and outpatient settings (€292.6 to 298.7), individual physiotherapy treatments (€34.9 to 35.7), occupational therapist treatments (€32.5 to 33.1), and psychosocial treatments (€87.0 to 88.9). Other resources and costs during the 12 months period were: specialist consultations (orthopaedic surgeon, hand surgeon, dermatologist, and orthotist; €158.7 to 320.0), radiography, (standard X-ray and Dual energy X-ray absorptiometry (DEXA) scanning; €52.9 to 128.1), and costs related to pharmacological therapy. For intravenous therapy, costs were proportional to the administered dose and administrations was used. Costs were calculated using 2009–2011 drug prices in Sweden (http://www.tlv.se).

### Sample size

Sample size for the study was calculated from a pre-trial power analysis, based on the previously published primary clinical outcome DAS28 score. This power analysis demonstrated that 95 patients would be a sufficient number to detect a clinically moderate difference between groups at a 5 % significance level with at least 90 % power. It was decided to include 107 patients to allow for a predicted 10 % drop out. With the previously established sample size of 95 patients and assuming the standard deviation (SD) being half of the costs, a difference in costs of 30 % could be analysed with a power of 80 %.

### Randomisation

**Fig. 1:**
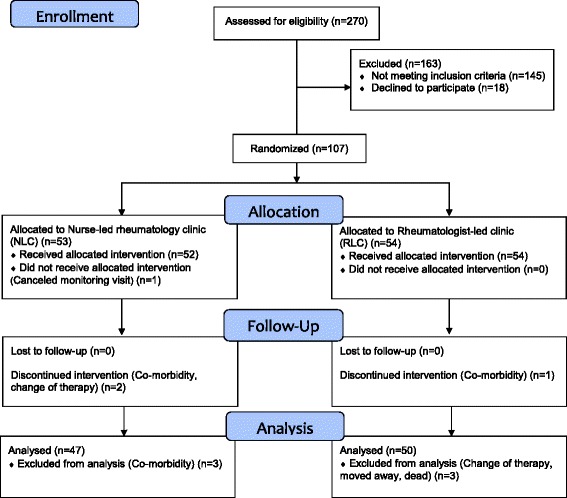
CONSORT Flow diagram of the recruitment and patients enrolled, allocated to nurse-led rheumatology clinic (NLC) or rheumatologist-led clinic (RLC), drop-outs and reasons for drop-out and the number of patients at 6 month follow-up and analysed after 12 months

Randomisation took the form of sealed envelopes containing assignment to one of the two groups. The envelopes were mixed and when a patient met the inclusion criteria an envelope was randomly picked. At inclusion, 18 patients decided not to participate, nine men and nine women (Fig. 1).

### Statistical analysis

Statistical analyses were performed using SPSS version 19.0 for Windows. Differences in direct costs of monitoring biological therapy and costs of rheumatology care between patients participating in the NLC compared to the RLC were analysed with independent sample *t*-test, using bootstrapping with 1000 iterations. *p* values < 0.05 were considered statistically significant.

### Ethics

The Regional Ethical Review Board at Lund University, Sweden, approved the trial (No. 2009/245; 2010/283). Patients received oral and written information about the RCT and their right to withdraw at any time and written informed consent was obtained from patients prior to inclusion in the trial. This trial conformed to the ethical principles for medical research on human beings set out in the declaration of Helsinki and fulfilled the four requirements on research: information, consent, confidentiality and safety of the participant [24]. This trial was registered at http://clinicaltrials.gov under the identification code NCT01071447.

## Results

From a total of 270 patients assessed by a rheumatologist, 125 met the inclusion criteria and were invited to participate in the trial. Of these, 107 agreed to take part and were randomly assigned to the NLC (*n* = 53) or to the RLC (*n* = 54). After 12 months 97 patients completed the trial (Fig. 1). At the inclusion the patients had a mean age of 55.4 years, disease duration of 16.7 years, and DAS28 was 2.1. Baseline demographics and disease characteristics of the patients are summarised in Table 1. Seventy-four percent of the patients had additional contacts with the rheumatology clinic besides the planned 6 and 12 months monitoring visits (Table 2).

**Table 1:** Baseline socio-demographic and clinical characteristics of patients in the nurse-led rheumatology clinic (NLC) and the rheumatologist-led clinic (RLC)

	NLC (*n* = 47)	RLC (*n* = 50)
Age (years)		
Mean	55.0 (12.3)	55.8 (13.2)
Range	34–81	21–77
Sex		
Female	26 (55)	28 (56)
Male	21 (45)	22 (44)
Civil status		
Co-habiting	35 (74)	39 (78)
Living alone	12 (26)	11 (22)
Education		
Compulsory comprehensive school	15 (32)	14 (28)
Upper secondary school	15 (32)	15 (30)
Undergraduate studies	17 (36)	21 (42)
Rheumatic disease		
Rheumatoid arthritis	25 (53)	35 (70)
Undifferentiated arthritis	1 (2)	3 (6)
Undifferentiated Spondyloarthritis	10 (21)	6 (12)
Peripheral Psoriatic arthritis	11 (23)	6 (12)
Disease duration (years)		
Mean	17.3 (10.9)	16.2 (12.1)
Range	1–44	1–52
Disease activity (DAS28)		
Mean	1.97 (0.67)	2.14 (0.71)
Range	0.61–3.20	0.53–3.06
Activity limitation (HAQ)		
Mean	0.45 (0.42)	0.63 (0.55)
Range	0.00–2.13	0.00–2.50
Health related Quality of life (Eq5D)		
Mean	0.77 (0.15)	0.73 (0.19)
Range	−0.01–1.00	0.09.1.00
Biologic therapies		
Adalimumab	7 (15)	18 (36)
Etanercept	13 (28)	18 (36)
Infliximab	27 (57)	14 (28)
Disease Modifying Anti-Rheumatic Drugs (DMARDs)		
Azathioprine	3 (6)	1 (2)
Hydroxychloroquine Sulfate	0 (0)	1 (2)
Methotrexate	29 (62)	27 (54)
Sulfasalazine	0 (0)	2 (4)
Methotrexate and Sulfasalazine	0 (0)	2 (4)

Values are reported as number (proportions), or mean values (SD)

**Table 2:** Comparison of resource use in monitoring of biological therapy in the nurse-led rheumatology clinic (NLC) and the rheumatologist-led clinic (RLC) over 12 months

	Numbers of contacts	NLC *n* = 47 (%)	RCL *n* = 50 (%)	Total *n* = 97
Any additional contacts	0	13 (28)	12 (24)	25 (26)
	≥1	34 (72)	38 (76)	72 (74)
Additional phone, nurse	0	26 (55)	27 (54)	53 (55)
	≥1 Range 1–7	21 (45)	23 (46)	44 (45)
Additional phone, rheumatologist	0	41 (87)	41 (82)	82 (85)
	≥1 Range 1–4	6 (13)	9 (18)	15 (15)
Additional rheumatologist visits	0	42 (89)	39 (78)	82 (85)
	≥1 Range 1–2	5 (11)	11 (22)	15 (15)
Addition cortisone inj. rheumatologist	0	36 (77)	42 (84)	78 (80)
	≥1 Range 1–3	11 (23)	8 (16)	19 (20)
Additional blood tests	0	36 (77)	27 (54)	63 (65)
	≥1 Range 1–12	11 (23)	23 (46)	34 (35)
Team rehabilitation inpatients (days)	0	47 (100)	46 (92)	93 (96)
	≥1 Range 15–24	0 (0)	4 (8)	4 (4)
Team Rehabilitation outpatients (days)	0	46 (98)	50 (100)	96 (99)
	≥1 Range 15–15	1 (2)	0 (0)	1 (1)
Physiotherapy	0	45 (96)	47 (94)	92 (95)
	≥1 Range 3–21	2 (4)	3 (6)	5 (5)
Occupational therapist	0	47 (100)	49 (98)	96 (99)
	≥1 Range 3–3	0 (0)	1 (2)	1 (1)
Psychosocial treatment	0	47 (100)	49 (98)	96 (99)
	≥1 Range 1–1	0 (0)	1 (2)	1 (1)
Specialist consulting	0	35 (74)	39 (78)	74 (76)
	≥1 Range 1–2	12 (26)	11 (22)	33 (34)
Radiography	0	32 (68)	34 (68)	66 (68)
	≥1 Range 1–5	15 (32)	16 (32)	31 (32)

### Primary outcome

The total annual rheumatology care costs including fixed monitoring, variable monitoring, rehabilitation, specialist consultations, radiography, and pharmacological therapy, generated €14107.7 per patient in the NLC compared with €16274.9 in the RCL (*p* = 0.004), giving a €2167.2 (13 %) lower annual cost for the NLC (Table 3).

**Table 3:** Comparison of resource use and rheumatology care cost (EURO) per patient in monitoring of biological therapy in the nurse-led rheumatology clinic (NLC) (*n* = 47) and the rheumatologist-led clinic (RLC) (*n* = 50) over 12 months

	Resource use in proportion NLC vs. RLC	Cost per^a^ unit in €	NLC Cost in €^a^ per patient Mean (SD)	RLC Cost in €^a^ per patient Mean (SD)	Difference^b^ Cost in €^a^Mean (95 % CI)	*p*	Percentage saving %
Primary outcome							
Total annual rheumatology care	1:1.2		14107.7 (3782.9)	16274.9 (3956.9)	−2167.2 (−3757.3 to −641.7)	0.004	13
Secondary outcomes							
Monitoring visit 6 months NLC/RLC	1:2.0	115.0–117.4/232.1–237.0	115.1 (0.5)	232.2 (0.7)	−117.2 (−117.4 to −117.0)	0.050	
Monitoring visit 12 months	1:1.0	232.1–237.0	235.7 (2.1)	235.2 (2.3)	0.5 (−0.4 to 1.4)	0.262	
Monitoring blood tests	1:1.0	15.4–15.5	30.9 (0.1)	30.9 (0.1)	0.0 (−0.0 to 0.0)	0.423	
Total fixed monitoring	1:1.3		381.7 (2.3)	498.3 (2.6)	−116.7 (−117.6 to −115.7)	0.001	23
Additional phone, nurse	1:1.8	6.2–6.4	3.9 (5.3)	6.9 (10.3)	−3.0 (−6.3 to 0.1)	0.060	
Additional phone, rheumatologist	1:1.9	16.9–17.2	2.5 (7.1)	4.8 (12.4)	−2.2 (−6.4 to 1.4)	0.287	
Additional rheumatologist visits	1:2.4	232.1–237.0	24.8 (72.7)	60.7 (122.9)	−35.9 (−76.2 to 0.7)	0.077	
Addition cortisone inj. to rheumatologist	1:0.7	174.1–177.7	63.1 (128.2)	45.6 (116.6)	17.5 (−31.6 to 64.4)	0.463	
Additional blood tests	1:3.8	1.2–30.7	5.5 (11.6)	21.1 (33.1)	−15.6 (−26.3 to −5.7)	0.014	
Total variable monitoring	1:1.4		99.8 (140.8)	139.1 (215.1)	−39.3 (−113.0 to 24.5)	0.292	
Total monitoring (fixed and variable)	1:1.3		481.5 (140.6)	637.4 (214.9)	−155.9 (−228.9 to −92.4)	0.001	24
Team rehabilitation inpatients (days)	0:79	406.2–418-8	0 (0)	647.5 (2251.7)	−647.5 (−1308.9 to −150.8)	0.086^c^	
Team rehabilitation outpatients (days)	15:0	292.6–298.7	93.4 (640.2)	0 (0)	93.4 (79.0 to 353.2)	0.135^d^	
Physiotherapy	1:0.4	34.9–35.7	26.2 (125.5)	10.9 (48.8)	15.3 (−17.8 to 55.9)	0.454^e^	
Occupational therapist	0:3.0	32.5–33.1	0 (0)	1.9 (13.8)	−1.9 (−7.6 to −1.7)	0.162^f^	
Psychosocial treatment	0:1.0	87.0–88.9	0 (0)	1.7 (12.3)	−1.7 (−6.8 to −1.5)	0.152^g^	
Total rehabilitation	1:5.5		119.6 (648.5)	662.1 (2248.0)	−542.5 (−1226.6 to 28.1)	0.142	82
Specialist consultations	1:1.0	158.7–320.0	76.0 (145.1)	78.1 (139.2)	−2.1 (−56.9 to 56.1)	0.949	
Radiography	1:1.6	52.9–128.1	38.6 (69.4)	60.8 (90.6)	−22.2 (−52.4 to 11.0)	0.162	
Pharmacological therapy	1:1.1		13376.7 (3608.0)	14821.2 (2909.4)	−1444.5 (−2740.1 to −278.5)	0.029	

^a^Costs are indexed to 2009–2011 when the resources were used and given in Euro

^b^Analysed with Bootstrap for Independent Samples Test: Unless otherwise noted, bootstrap results are based on 1000 bootstrap samples

^c^Based on 978 samples

^d^Based on 616 samples

^e^Based on 994 samples

^f^Based on 633 samples

^g^Based on 619 samples

### Secondary outcomes

The fixed costs were directly related to the differences in cost for a nurse visit compared to a visit to the rheumatologist (Table 3). The monitoring generated variable costs of €99.8 per patient in the NLC compared with €139.1 in the RCL (*p* = 0.292). There were no significant differences in the individual variables additional telephone calls to a rheumatology nurse (1:1.8; *p* = 0.060), additional telephone calls to a rheumatologist (1:1.9; *p* = 0.287), additional rheumatologist visits (1:2.4; *p* = 0.077), cortisone injections in addition to regular rheumatologist monitoring visits (1:0.7; *p* = 0.463). There was a significant difference between the groups regarding blood tests (1:3.9; *p* = 0.014). The total annual monitoring costs, fixed costs and variable costs, generated €481.5 per patient in the NLC compared with €637.4 for monitoring in the RCL (*p* = 0.001), generating a €155.9 (24 %) lower annual cost for the NLC A total of 11 patients had inpatient or outpatient rehabilitation contacts generating costs during the 12 months follow-up (Table 2). The annual cost of rehabilitation per patient monitored by the NLC was €119.6 compared with €662.1 for monitoring by the RLC (*p* = 0.142). There were no significant differences between the groups in the individual variables team rehabilitation in inpatients settings (*p* = 0.086), outpatient settings (*p* = 0.135), individual physiotherapy treatments (*p* = 0.454), occupational therapist treatments (*p* = 0.162), and psychosocial treatments (*p* = 0.152) (Table 3).

There were no significant differences between the groups in the costs related to specialist consultations (*p* = 0.949) and radiography (*p* = 0.162). There was a significant difference between the groups in pharmacological therapy and cost related to this (*p* = 0.029) (Table 3).

## Discussion

This is a study comparing the differences in resources and costs when substituting a rheumatologist with a rheumatology nurse in monitoring patients with stable CIA undergoing biological therapy. Replacing one of the two annual rheumatologist monitoring visits by a nurse-led monitoring visit resulted in no additional contacts to the rheumatology clinic, but rather a decrease in use of resources and a reduction of costs. This reduction in use of resources and lower costs were not related to any differences between the groups in clinical outcomes as previously reported from this RCT [20].

In rheumatology care, there are only a few studies evaluating the cost-effectiveness of an NLC and almost only in conventional DMARD therapy. These studies reveal that NLCs are more cost-effective regarding cost and disease-related dimensions such as DAS28 or Eq5D, but not clearly in relation to quality-adjusted life years [17, 18, 25]. The present study demonstrated lower resource use and costs when monitoring biological therapy by an NLC compared to an RLC over a 12 month follow-up period. This is mainly due to the fixed monitoring costs, where a visit to rheumatologist is more costly than a visit to a rheumatology nurse. The result also suggests, although not significant, that patients monitored in the NLC in comparison with the RLC had lower use of variable monitoring resources and costs. This may be due to the visit to the NLC with a PCC approach being focused on the patient’s resources and needs. Patients’ narratives create a common understanding of the illness experience, which, together with the symptoms of the disease, provide the nurse with a good foundation for discussing and planning care and treatment together with the patient [23]. This is consistent with previous research showing that an NLC leads to fewer additional contacts with healthcare services [26]. PCC increases patients’ confidence in their own ability and patients become autonomous and independent [27]. Research has shown that PCC in various fields of inpatient care has led to a reduction in the length of the hospital stay by up to 70 % and reduced costs without a negative impact on health-related quality of life [12, 13, 28, 29]. The present study showed more additional blood tests in the RLC. These were predominantly routine test and not expensive special test, and they were ordered despite the fact that the patients had a stable CIA and were monitored every six months. The monitoring visit at the NLC included a dialogue around the pharmacological therapy in terms of administration, adherence, side effects and blood tests. The finding in the present study is in line with that patients with knowledge about their disease and its treatment and monitoring, including blood tests, have been reported to use less health care resources [30]. For annual inpatient rehabilitation there was a non-significant lower cost in the NLC group compared with the RLC group. This was however based on only a small number of patients receiving rehabilitation during the 12 months. Previous studies have reported that rheumatology nurses often refer patients to individual team members based on individual needs [31, 32].

This study also evaluated differences in pharmacological therapy and costs related to this. There was a lower cost in the NLC compared to the RLC. This was due to a greater proportion of patients in the RLC treated with subcutaneous biological therapy, being more expensive than intravenous biological therapy in Sweden (http://www.tlv.se). Other methods of calculating the cost of biological therapy have shown that the annual cost per patient for intravenous infusions is more expensive than for subcutaneous injections [33]. Due to the increasing total rheumatology care costs [19] and the effectiveness of the expensive biological therapies in rheumatology care [34] the present study supports the EULAR recommendations, which argue that interventions and monitoring by nurses could contribute to cost savings in comprehensive disease management [1]. The result is important because it suggests that the annual resource use and costs are lower when monitoring biological therapy in an NLC, based on PCC, in comparison with monitoring in an RLC. The patients were monitored effectively by two annual visits [20], which differs from previous studies on patients undergoing conventional DMARD treatment, where NLCs are based on frequent visits to the nurse, usually every 3 months or more often [14–17]. When the cost of the rheumatology care can be reduced by replacing rheumatologists with rheumatology nurses in monitoring patients who have a low disease activity or remission, resources can be reallocated to patients who have a high disease activity and do not respond to medical treatment. These patients may need a more tight control of their disease with frequent visits to the rheumatologist, which is an effective strategy in patients with RA [35] as well as in SpA [36], or treatment from a multidisciplinary team [37, 38]. Research demonstrates that treating to the target of remission in early rheumatoid arthritis is cost-effective [39].

This study suggests that implementation of NLC in rheumatology care should be considered as it could save resources and costs, with no differences in disease activity or activity limitations as shown in previous studies [14, 16, 17, 20]. A regular contact with a rheumatology nurse as a complement to a rheumatologist provides added value to the rheumatology care [40]. The rheumatology nurse listens attentively and is sensitive in their conversation so the patients dare to open up and experience confirmation about their illness experience and life situation [21]. An NLC adds value to the rheumatology care in terms of increased satisfaction [15–17, 41] and empowering patients to achieve a higher level of confidence in their own abilities [42] and participation. This is consistent with the results from the present study. Patients experience participation due to exchange of information, dialogue and respect of their own knowledge and skills [21]. It is important for patients to be seen as individuals [21, 42–44] and a PCC with a holistic approach is essential in the management of patients with CIA [21, 43], which may have influenced the tendency towards a higher health related quality of life in the NLC. There are still, however, some challenges especially from the rheumatologists, who doubt the nurses’ knowledge, but also from patients, who express fear of losing contact with the rheumatologist [45]. Patients experience, however, a sense of security and describe rheumatology nurses as competent and skilled and point to the nurses’ high level of knowledge [21, 42–44].

The study has both strengths and limitations. The strengths are the design based on an RCT, the inclusion of all rheumatology care, and that 90.6 % of the randomly assigned participants had complete data and fulfilled the study. A limitation is that this was a single center trial, but with patients from three regions in Sweden. Another limitation is that it focuses on the direct costs of rheumatology care and does not include indirect costs or savings outside healthcare services or for the patients themselves. There could also be additional costs or savings in other healthcare areas but this is not covered by the present study.

## Conclusions

Patients with stable CIA undergoing biological therapy can be monitored with reduced resource use and lower annual costs by an NLC, based on PCC, compared to an RLC, with no difference in clinical outcomes. The intervention with an NLC could free resources for rheumatologists for more intensive monitoring of patients early in the course of the disease or patients with high disease activity. With the rapid development of biological therapies there is a need of more extensive and comprehensive studies of in resource use and cost savings of NLC in monitoring these therapies.

## Abbreviations

CIA: Chronic inflammatory arthritis
DAS28: Disease activity score 28
DMARD: Disease-modifying anti-rheumatic drug
EULAR: European league against rheumatism
NLC: Nurse-led rheumatology clinic
PCC: Person-centred care
RA: Rheumatoid arthritis
RCT: Randomised controlled trial
RLC: Rheumatologist-led clinic
SPA: Spondyloarthritis
